# Crime-scene and offender characteristics in conventional and nonconventional stranger homicides committed by male offenders in Sweden

**DOI:** 10.1177/00258024241255779

**Published:** 2024-05-27

**Authors:** Sara Rodre, Joakim Sturup, Thomas Masterman

**Affiliations:** 1 Department of Forensic Psychiatry, 27104 National Board of Forensic Medicine, Huddinge, Sweden; 2 Department of Clinical Neuroscience, 27106 Karolinska Institutet, Huddinge, Sweden; 3 7664 Swedish Police Authority, Stockholm, Sweden

**Keywords:** Stranger homicide, crime-scene characteristics, offender characteristics, severe mental disorder, mental illness, act of madness

## Abstract

In Sweden, from 1990 to 2013, most homicides occurred between family members, friends or acquaintances: the annual rate of incidents between unacquainted offenders and victims ranged between 8% and 13%. In the majority of these “stranger homicides,” three common motives, as defined by the precipitating event, could be identified: homicides resulting from a spontaneous altercation; homicides committed in the context of a robbery or burglary; and homicides committed in the context of a gangland conflict. The remaining minority—with uncommon or indiscernible motives—could, nonetheless, be categorized according to their nonconventional distinguishing feature: homicides characterized by the offender's ostensibly mentally aberrant behavior; homicides committed in the context of a hate offense or politically motivated offense; homicides committed in the context of a sexual offense; and homicides committed in the context of a mass killing or series of homicides. In this registry-based study of 224 incidents, “conventional” stranger homicides, defined by their commonplace motive, were compared with “nonconventional” stranger homicides, defined by their lack of such motive. The former were more often committed with an accomplice, against a male victim, whereas the latter were more often committed in a public place, after contact initiated by the offender. In the latter, offenders were less often intoxicated at the time of the offense and more often adjudged to suffer from a severe mental disorder. The subcategory of nonconventional stranger homicides characterized by the offender's ostensibly mentally aberrant behavior corresponded largely to both the archetypal stranger-homicide construct and the popular notion “act of madness.”

## Introduction

In a recent research report from the Nordic countries,^1^ homicide is defined as “an intentional criminal act of violence by one or more human beings resulting in the death of one or more other human beings.” In Sweden, between the years 1990 and 2013, the overwhelming majority of homicides occurred between family members, friends or acquaintances, while the annual rate of incidents in which the offender and victim were unacquainted ranged between 8% and 13%.^2^ For purposes of comparison, in a sample of 48,885 homicide incidents committed in the United States between 2003 and 2015, stranger victims were involved in 14% of mass homicides and 20% of homicides with fewer victims^3^; similarly, in a sample of 539 homicides committed in Japan between 1998 and 2002, 17% of incidents involved stranger victims.^4^ In turn, in the majority of incidents involving unacquainted offenders and victims during the period, three common motives—as defined by the precipitating event—could be identified: homicides resulting from a spontaneous altercation; homicides committed in the context of a robbery or burglary; and homicides committed in the context of a gangland conflict. The remaining minority of cases—in which motives were either uncommon or indiscernible—could, nonetheless, be categorized according to their nonconventional distinguishing feature: homicides characterized by the offender's ostensibly mentally aberrant behavior at the time of the offense; homicides committed in the context of a hate offense or politically motivated offense; homicides committed in the context of a sexual offense; and homicides committed in the context of a mass killing or series of homicides.

Arguably, it is these latter, nonconventional homicides that the concept “stranger homicide” was originally introduced to denote. Indeed, in the opening paragraph of his influential review of the concept, Riedel^5^ wrote: “Stranger violence represents one of the most frightening forms of criminal victimization. […] It is suggested that people fear the unknown person who commits an unpredictable and violent attack on a vulnerable and innocent citizen going about routine daily activities. The perceptions that the attacker is indiscriminate in his selection of the victim and that the victim can do little to avoid attack or protect himself also elicit fear in society.” Similarly, in the same themed issue of *The Journal of Criminal Law and Criminology*, Langevin and Handy^6^ highlighted the incomprehensibility of lethal violence between total strangers, in contrast to “understandable” homicides committed within romantic involvements or rivalries.

A handful of recent studies have focused on a subset of stranger homicides thus conceived, stranger sexual homicides. In a descriptive study of 81 sexual homicides committed by adult male offenders on adult female victims in the United Kingdom between 1970 and 2010, 52.5% of homicides occurred on a Friday, Saturday or Sunday, and 68% occurred between the hours of 6 p.m. and 3 a.m.^7^; based on the observation that the consumption of alcohol and drugs is a common activity on evenings and weekends—as well as on previous research on alcohol and drug use in rape and homicides offenders and victims—the study's authors remarked that some adult male-on-female stranger sexual homicides appear to be “impulsive, opportunistic acts undertaken by intoxicated men against vulnerable intoxicated women.” At the same time, a subsequent statistical analysis of the correlational structure of crime-scene actions in the same dataset uncovered dimensional heterogeneity in the sample, in the form of four identifiable behavioral subthemes, which the authors designated rape, impersonal sexual assault, overkill and control.^8^ Further, the authors of a study comparing 463 nonsexual homicides and 173 sexual homicides committed in France between 1979 and 2014 concluded that differences in modus operandi between the two groups were in part attributable to the fact that sexual homicides are typically committed against victims who are strangers to the offender^9^; among other differences, sexual homicides were almost four times more likely to be committed by means of surprise attack and more than twice as likely to be committed in the presence of witnesses. At the same time, in a more recent study of 662 nonserial sexual homicides committed in Canada and France between 1948 and 2018—involving 562 female victims and 100 male victims—the same authors found that male victims were less often assaulted by stranger offenders.^10^

Nonetheless, in traditional criminological surveys of the offender–victim relationship, homicidal strangers are most often all lumped into one category; thus, offenses fitting the description of the archetypal, intuitive stranger-homicide construct—incomprehensible acts of violence that, unpredictably and indiscriminately, target unsuspecting citizens—are there greatly outnumbered by more conventional stranger-homicide incidents.^11–13^ In fact, in at least one study,^14^ stranger homicides are defined, still less intuitively, as homicides in which the identity of the offender was, at the time the crime was discovered, unknown to the police. By contrast, the archetypal construct corresponds more closely to the FBI profile of the “motiveless homicide”; in the bureau's crime-classification manual, such homicides are characterized as irrational acts committed against randomly chosen victims in a public location.^15^ In a study based on a French national police database of extrafamilial homicides committed between 1970 and 2018, about two-fifths of motiveless homicides thus defined were committed against stranger victims, and about four-fifths were committed by means of con, blitz or surprise approach.^16^

As a category, the nonconventional stranger homicides committed in Sweden between 1990 and 2013 in the present dataset thus bear similarities to both the archetypal stranger-homicide construct and the FBI's motiveless-homicide profile; the category also overlaps, conceptually and chronologically, with the notion—current, during the period, in the Swedish popular press—of an “act of madness” (*vansinnesdåd*). As Höijer and Rasmussen^17^ point out, the term “act of madness” has been used by journalists to describe unforeseeable violent events occurring in public spaces that are carried out by ostensibly mentally ill perpetrators with no personal relationship with their victims.

Like “act of madness,” the category nonconventional stranger homicide in the present dataset consists primarily of unprovoked and seemingly motiveless crimes; further, many nonconventional stranger homicides with identifiable motives are either carried out suddenly and publicly, such as political assassinations and certain mass shootings, or preceded by a sudden and public abduction, as in many sexual and serial homicides. (On the other hand, admittedly, an unprovoked attack need not prove lethal for it, in the press, to be designated an “act of madness.”)

In the present study, informed by both the archetypal construct of stranger homicide and the popular notion “act of madness,” we attempted to investigate the phenomenon of lethal violence between strangers by differentiating conventional stranger homicides, subcategorized by their precipitating event, from nonconventional stranger homicides, subcategorized by their distinguishing feature. Our starting point was a registry of all homicides committed in Sweden during the study period that included variables reflecting crime-scene and offender characteristics, such as antecedent incidents ranked by degree of offender initiation and the presence or absence, in the offender, of a severe mental disorder. In accordance with the archetypal construct and the popular notion described above, we hypothesized, first, that, in nonconventional stranger homicides, offenses would more often have been initiated by the offenders themselves; and, second, that, upon subsequent forensic-psychiatric evaluation, perpetrators of nonconventional stranger homicides would more often have been adjudged to be suffering from a severe mental disorder.

## Methods

### Incidents and offenders

Data concerning all homicide incidents in Sweden are routinely cataloged in a nationwide registry administered by the Swedish National Council for Crime Prevention. According to the Swedish penal code, homicide is defined as murder, voluntary manslaughter, involuntary manslaughter by means of assault and infanticide. From the registry, from a total of 2123 homicide incidents committed between 1 January 1990 and 31 December 2013, in which the offender or offenders had been convicted or otherwise identified, we retrieved all incidents in which offenders and victims, according to the registry, had been unacquainted with one another prior to the offense (*n *= 238). In cases in which the same offender had been involved in multiple homicide incidents (*n *= 7), only data concerning the chronologically first incident were retained in the dataset. For homicide incidents in which more than one offender had been convicted or identified (*n *= 76) or for which there had been more than one victim (*n *= 15), only primary offenders and victims, as designated in the registry, were retained in the dataset.

Thus, the remaining dataset contained information on 231 homicide incidents, including, for each incident, information concerning one unique offender and one unique victim. In the registry, under the heading “type of homicide,” the Swedish National Council for Crime Prevention employs a hierarchical scheme whereby each homicide incident is assigned to a single subcategory. Because the purpose of the study was to compare conventional stranger homicide with nonconventional stranger homicide, incidents subcategorized by their commonplace precipitating event were included in the former group, whereas incidents subcategorized by their unusual distinguishing feature were included in the latter. The precipitating event comprised three subcategories: spontaneous altercation; robbery or burglary; and gangland conflict. Distinguishing feature comprised four subcategories: offender's ostensibly mentally aberrant behavior; committed in the context of a hate offense or politically motivated offense; committed in the context of a sexual offense; and committed in context of a mass killing or series of homicides. At this stage, three additional incidents were excluded on account of missing data regarding the homicide incident's precipitating event or distinguishing feature. The paucity of female offenders in the remaining dataset (*n *= 4) precluded stratification by sex; thus, after the exclusion of female offenders, the final dataset consisted of 224 homicide incidents committed by male offenders.

### Crime-scene and offender characteristics

Based on information available in the registry, the following crime-scene characteristics were compared between conventional-stranger-homicide and nonconventional-stranger-homicide groups: month of the offense, ordered by daily temperature in Sweden; whether the offense had been committed between Friday evening and Monday morning; time of the offense, ordered by degree of daylight; whether the offense had been committed together with at least one accomplice; sex of the victim; the age of the victim; whether the victim had been born abroad; whether the victim had been intoxicated at the time of the offense; site of the offense, ordered by degree of seclusion; antecedent incident, ordered by degree of offender initiation; and method of violence, ordered by degree of intimacy. In addition, the following offender characteristics were compared between groups: age of the offender; whether the offender had been born abroad; whether the offender had been intoxicated at the time of the offense; the time elapsed between the police becoming aware of the offense and the apprehension of the offender; whether the offender had at least one previous criminal conviction; and whether the offender, upon forensic-psychiatric evaluation, had been adjudged to be suffering from a severe mental disorder.

### Statistical analysis

For categorical variables, frequencies in the conventional-stranger-homicide and nonconventional-stranger-homicide groups were compared using Pearson's chi-squared test or a chi-squared test for trend. For the continuous—and in the dataset nonnormally distributed—variables age of the victim and age of the offender, differences in ranked ages in the respective groups were compared using the Mann-Whitney *U* test. All tests were performed using IBM SPSS Statistics for Windows, Version 27.0 (Armonk, NY: IBM Corporation). To correct for multiple comparisons, two-sided probability values less than the quotient of 0.05 divided by 17 (the number of independent tests performed)—approximately 0.00294—were considered statistically significant.

### Ethics

The study was approved by the Regional Ethical Review Board in Stockholm (Ref No. 2014/749-31/5).

## Results

### Crime-scene characteristics

In Tables 1 and 2, crime-scene characteristics are presented for 164 conventional stranger homicides and 60 nonconventional stranger homicides committed by male offenders; in the former group, homicides are subcategorized by precipitating event, and in the latter group by distinguishing feature. The majority of conventional stranger homicides had been committed during the warmest six months of the year, whereas the majority of nonconventional stranger homicides had been committed during the coldest six months of the year; the difference, however, was not statistically significant, and, within each group, there was little variation between subcategories with regard to percentages. In each group, approximately half of the homicides had been committed during the weekend (defined as beginning on Friday evening and ending on Monday morning); however, more than two-thirds of nonconventional stranger homicides in which the distinguishing feature was either a hate offense, a politically motivated offense or a sexual offense had been committed during the weekend. About two-fifths of nonconventional stranger homicides had been committed during the daytime, compared to about one-fifth of conventional stranger homicides; the difference was not statistically significant, but in the former group, percentages of homicides committed in the daytime varied between subcategories, from nearly 60% in incidents characterized by the offender's ostensibly mentally aberrant behavior to around 15% in incidents committed in the context of a sexual offense. Conventional stranger homicides were four times more likely to have been committed with at least one accomplice than nonconventional stranger homicides, a difference that was statistically significant; in fact, whereas over 70% of homicides precipitated by gangland conflicts had involved at least one accomplice, homicides characterized by the offender's ostensibly mentally aberrant behavior or committed in the context of a sexual offense had been, without exception, committed alone.

**Table 1.** Crime-scene and offender characteristics in 164 conventional and 60 nonconventional stranger homicides committed by male offenders in Sweden from 1990 to 2013, subcategorized by precipitating event or distinguishing feature. (In the case of missing data, denominators are indicated in brackets.)

	Conventional stranger homicides, by precipitating event			Nonconventional stranger homicides, by distinguishing feature			
	Spontaneous altercation	Robbery or burglary	Gangland conflict	Offender's aberrant behavior	Committed in the context of hate offense or politically motivated offense	Committed in the context of sexual offense	Committed in context of mass killing or series of homicides
Crime-scene characteristics							
Number of incidents	96	54	14	26	16	13	5
Month of offense, by ascending average daily temperature in Sweden							
December, January or February, *n* (%)	22 (22.9%)	11 (20.4%)	5 (35.7%)	4 (15.4%)	5 (31.3%)	5 (38.5%)	2 (40%)
September, October or November, *n* (%)	17 (17.7%)	12 (22.2%)	1 (7.1%)	11 (42.3%)	4 (25.9%)	3 (23.1%)	1 (20%)
March, April or May, *n* (%)	24 (25%)	14 (25.9%)	4 (28.6%)	5 (19.2%)	2 (12.5%)	0 (0%)	0 (0%)
June, July or August, *n* (%)	33 (34.4%)	17 (31.5%)	4 (28.6%)	6 (23.1%)	5 (31.3%)	5 (38.5%)	2 (40%)
Offense committed between Friday evening and Monday morning, *n* (%)	52 (58.4%) (*n* = 89)	17 (33.3%) (*n* = 51)	5 (38.5%) (*n* = 13)	10 (41.7%) (*n* = 24)	11 (78.6%) (*n* = 14)	9 (69.2%)	2 (40%)
Time of offense, by ascending degree of daylight	(*n* = 85)	(*n* = 52)	(*n* = 12)	(*n* = 24)	(*n* = 14)		
Nighttime, *n* (%)	51 (60%)	17 (32.7%)	6 (50%)	4 (16.7%)	6 (42.9%)	8 (61.5%)	1 (20%)
Evening, *n* (%)	25 (29.4%)	20 (38.5%)	2 (16.7%)	6 (25%)	4 (28.6%)	3 (23.1%)	2 (40%)
Daytime, *n* (%)	9 (10.6%)	15 (28.8%)	4 (33.3%)	14 (58.3%)	4 (28.6%)	2 (15.4%)	2 (40%)
Committed with accomplice(s), *n* (%)	28 (29.2%)	28 (51.9%)	10 (71.4%)	0 (0%)	5 (31.3%)	0 (0%)	1 (20%)
Male victim, *n* (%)	91 (94.8%)	46 (85.2%)	13 (92.9%)	17 (65.4%)	16 (100%)	0 (0%)	4 (80%)
Age of victim in years, median (range)	29 (16–83) (*n* = 85)	50 (17–91) (*n* = 50)	28.5 (8–54) (*n* = 12)	53 (5–88) (*n* = 25)	35 (17–73) (*n* = 15)	27 (15–91)	37 (22–65)
Victim born abroad, *n* (%)	17 (19.8%) (*n* = 86)	10 (18.9%) (*n* = 53)	7 (53.8%) (*n* = 13)	3 (12.5%) (*n* = 24)	5 (31.3%)	0 (0%)	3 (60%)
Victim intoxicated at time of offense, *n* (%)	64 (72.7%) (*n* = 88)	10 (20.0%) (*n* = 50)	1 (11.1%) (*n* = 9)	5 (20.8%) (*n* = 24)	7 (53.8%) (*n* = 13)	5 (38.5%)	0 (0%) (*n* = 3)
Site of offense, by ascending degree of seclusion							
Public place, in presence of witness(es), *n* (%)	75 (78.1%)	15 (27.8%)	4 (28.6%)	12 (46.2%)	5 (31.3%)	0 (0%)	1 (20%)
Public place, in absence of witness(es), *n* (%)	11 (11.5%)	14 (25.9%)	4 (28.6%)	3 (11.5%)	5 (31.3%)	2 (15.4%)	1 (20%)
In home, in presence of witness(es), *n* (%)	3 (3.1%)	5 (9.3%)	3 (21.4%)	3 (11.5%)	1 (6.3%)	0 (0%)	1 (20%)
Country road, park of forest, in presence of witness(es), *n* (%)	2 (2.1%)	4 (7.4%)	0 (0%)	2 (7.7%)	0 (0%)	0 (0%)	0 (0%)
Country road, park of forest, in absence of witness(es), *n* (%)	1 (1.0%)	3 (5.6%)	1 (7.1%)	2 (7.7%)	0 (0%)	8 (61.5%)	1 (20%)
In home, in absence of witness(es), *n* (%)	4 (4.2%)	13 (24.1%)	2 (14.3%)	4 (15.4%)	5 (31.3%)	3 (23.1%)	1 (20%)
Antecedent incident, by ascending degree of offender initiation	(*n* = 92)	(*n* = 48)	(*n* = 10)	(*n* = 20)	(*n* = 11)	(*n* = 12)	
Victim initiates contact, *n* (%)	13 (14.1%)	13 (27.1%)	0 (0%)	1 (5%)	2 (18.2%)	0 (0%)	0 (0%)
Mutual conflict between offender and victim, *n* (%)	66 (71.7%)	11 (22.9%)	5 (50%)	7 (35%)	4 (36.4%)	2 (16.7%)	1 (20%)
Offender initiates contact, *n* (%)	5 (5.4%)	1 (2.1%)	0 (0%)	1 (5%)	1 (9.1%)	5 (41.7%)	0 (0%)
Surprise attack by offender, *n* (%)	8 (8.7%)	23 (47.9%)	5 (50%)	11 (55%)	4 (36.4%)	5 (41.7%)	4 (80%)
Method of violence, by ascending degree of intimacy	(*n* = 94)	(*n* = 51)		(*n* = 25)			
Firearm, vehicle or arson, *n* (%)	12 (12.8%)	12 (23.5%)	11 (78.6%)	2 (8%)	1 (6.3%)	0 (0%)	5 (100%)
Knife, axe or other sharp object, *n* (%)	46 (48.9%)	23 (45.1%)	2 (14.3%)	15 (60%)	12 (75%)	4 (30.8%)	0 (0%)
Battery (with or without blunt object), *n* (%)	34 (36.2%)	13 (25.5%)	1 (7.1%)	5 (20%)	1 (6.3%)	2 (15.4%)	0 (0%)
Strangulation, suffocation or drowning, *n* (%)	2 (2.1%)	3 (5.9%)	0 (0%)	3 (12%)	2 (12.5%)	7 (53.8%)	0 (0%)
Offender characteristics							
Age in years, median (range)	22 (15–58)	25 (15–48)	25 (19–38)	29.5 (16–66)	21.5 (16–29)	29 (19–38)	30 (24–38)
Born abroad, *n* (%)	25 (26.9%) (*n* = 93)	23 (43.4%) (*n* = 53)	6 (46.2%) (*n* = 13)	8 (33.3%) (*n* = 24)	8 (50.0%)	5 (38.5%)	1 (20.0%)
Intoxicated at time of offense, *n* (%)	76 (81.7%) (*n* = 93)	30 (61.2%) (*n* = 49)	4 (36.4%) (*n* = 11)	12 (48%) (*n* = 25)	6 (50.0%) (*n* = 12)	4 (30.8%) (*n* = 13)	2 (40%)
Time between police's awareness of offense and apprehension	(*n* = 91)	(*n* = 51)	(*n* = 13)		(*n* = 12)		
In conjunction with offense, *n* (%)	23 (25.3%)	1 (2.0%)	2 (15.4%)	5 (19.2%)	3 (25%)	0 (0%)	1 (20%)
Within 48 h, *n* (%)	36 (39.6%)	25 (49.0%)	1 (7.7%)	15 (57.7%)	4 (33.3%)	3 (23.1%)	0 (0%)
Within 1 month, *n* (%)	21 (23.1%)	16 (31.4%)	2 (15.4%)	4 (15.4%)	4 (33.3%)	4 (30.8%)	0 (0%)
After 1 month, *n* (%)	11 (12.1%)	9 (17.6%)	8 (61.5%)	2 (7.7%)	1 (8.3%)	6 (46.2%)	4 (80%)
Previous criminal conviction(s), *n* (%)	74 (81.3%) (*n* = 91)	41 (80.4%) (*n* = 51)	10 (71.4%)	14 (58.3%) (*n* = 24)	7 (50%) (*n* = 14)	11 (84.6%)	3 (60%)
Severe mental disorder, *n* (%)	2 (2.1%)	1 (1.9%)	1 (7.1%)	20 (76.9%)	4 (25%)	2 (15.4%)	0 (0%)

**Table 2.** Comparison of crime-scene and offender characteristics in 164 conventional and 60 nonconventional stranger homicides committed by male offenders in Sweden from 1990 to 2013. (In the case of missing data, denominators are indicated in brackets. All probability values are two-sided.)

	Conventional stranger homicides	Nonconventional stranger homicides	Comparison of frequencies or distributions between conventional and nonconventional stranger homicides
Crime-scene characteristics			
Number of incidents	164	60	
Month of offense, by ascending average daily temperature in Sweden			
December, January or February, *n* (%)	38 (23.2%)	16 (26.7%)	
September, October or November, *n* (%)	30 (18.3%)	19 (31.7%)	χ2(1, *N *= 224) = 1.74, *p* = .19*
March, April or May, *n* (%)	42 (25.6%)	7 (11.7%)	
June, July or August, *n* (%)	54 (33.0%)	18 (30%)	
Offense committed between Friday evening and Monday morning, *n* (%	74 (48.4%) (*n* = 153)	32 (57.1%) (*n* = 56)	χ2(1, *n* = 209) = 1.26, *p* = .26†
Time of offense, by ascending degree of daylight	(*n* = 149)	(*n* = 56)	
Nighttime, *n* (%)	74 (49.7%)	19 (34.0%)	
Evening, *n* (%)	47 (31.5%)	15 (26.8%)	χ2(1, *n* = 205) = 8.14, *p* = .0043*
Daytime, *n* (%)	28 (18.8%)	22 (39.3%)	
Committed with accomplice(s), *n* (%)	66 (40.2%)	6 (10%)	χ2(1, *N* = 224) = 18.42, *p* < .0001†
Male victim, *n* (%)	150 (91.5%)	37 (61.7%)	χ2(1, *N* = 224) = 28.28, *p* < .000001*
Age of victim in years, median (range)	33 (8–91) (*n* = 147)	38.5 (5–91) (*n* = 58)	*U* = 4412, *p* = .70‡
Victim born abroad, *n* (%)	34 (22.4%) (*n* = 152)	11 (19%) (*n* = 58)	χ2(1, *n* = 210) = 0.29, *p* = .59†
Victim intoxicated at time of offense, *n* (%)	75 (51.0%) (*n* = 147)	17 (32.1%) (*n* = 53)	χ2(1, *n* = 200) = 5.63, *p* = .018†
Site of offense, by ascending degree of seclusion			
Public place, in presence of witness(es), *n* (%)	94 (57.3%)	18 (30%)	
Public place, in absence of witness(es), *n* (%)	29 (17.7%)	11 (18.3%)	
In home, in presence of witness(es), *n* (%)	11 (6.7%)	5 (4.3%)	χ2(1, *N* = 224) = 16.57, *p* < .0001*
Country road, park of forest, in presence of witness(es), *n* (%)	6 (3.7%)	2 (3.3%)	
Country road, park of forest, in absence of witness(es), *n* (%)	5 (3.0%)	11 (18.3%)	
In home, in absence of witness(es), *n* (%)	19 (11.6%)	13 (21.7%)	
Antecedent incident, by ascending degree of offender initiation	(*n* = 150)	(*n* = 48)	
Victim initiates contact, *n* (%)	26 (17.3%)	3 (6.3%)	
Mutual conflict between offender and victim, *n* (%)	82 (54.7%)	14 (29.2%)	χ2(1, *n* = 198) = 17.10, *p* < .0001*
Offender initiates contact, *n* (%)	6 (4%)	7 (14.6%)	
Surprise attack by offender, *n* (%)	36 (24%)	24 (50%)	
Method of violence, by ascending degree of intimacy	(*n* = 159)	(*n* = 59)	
Firearm, vehicle or arson, *n* (%)	35 (22%)	8 (13.6%)	
Knife, axe or other sharp object, *n* (%)	71 (44.7%)	31 (52.5%)	χ2(1, *n* = 218) = 4.09, *p* = .043*
Battery (with or without blunt object), *n* (%)	48 (30.2%)	8 (13.6%)	
Strangulation, suffocation or drowning, *n* (%)	5 (3.1%)	12 (20.3%)	
Offender characteristics			
Age in years, median (range)	23 (15–58)	26 (16–66)	*U* = 5817, *p* = .37‡
Born abroad, *n* (%)	54 (34.0%) (*n* = 159)	22 (37.9%) (*n* = 58)	χ2(1, *n* = 217) = 0.29, *p* = .59†
Intoxicated at time of offense, *n* (%)	110 (71.9%) (*n* = 153)	24 (43.6%) (*n* = 55)	χ2(1, *n* = 208) = 14.10, *p* < .001†
Time between police's awareness of offense and apprehension	(*n* = 155)	(*n* = 56)	
In conjunction with offense, *n* (%)	26 (16.8%)	9 (16.1%)	
Within 48 h, *n* (%)	62 (40%)	22 (39.3%)	χ2(1, *n* = 211) = 0.22, *p* = .64*
Within 1 month, *n* (%)	39 (25.2%)	12 (21.4%)	
After 1 month, *n* (%)	28 (18.1%)	13 (23.2%)	
Previous criminal conviction(s), *n* (%)	125 (80.1%) (*n* = 156)	35 (62.5%) (*n* = 56)	χ2(1, *n* = 212) = 6.92, *p* = .0085†
Severe mental disorder, *n* (%)	4 (2.4%)	26 (43.3%)	χ2(1, *N* = 224) = 63.34, *p* < .00000000000001†

*chi-squared test for trend.

†Pearson's chi-squared test.

‡Mann-Whitney *U* test.

As seen in the tables, homicide victims in both groups were predominantly male; however, owing largely to the absence of male victims in incidents committed in the context of a sexual offense, male victims were significantly more common in conventional stranger homicides. The median age of victims was five and a half years higher in nonconventional stranger homicides than in conventional stranger homicides—38.5 years versus 33 years—but the distribution of ranked ages did not differ significantly between groups. The percentage of victims born abroad was also similar, at about 20% in both groups. About half of the conventional-stranger-homicide victims—including over 70% of victims killed in spontaneous altercations—had been intoxicated at the time of the offense, compared to about one-third of nonconventional-stranger-homicide victims; the difference, however, was not statistically significant.

The site of the offense was ranked according to the degree of seclusion, as seen in the tables. Three-quarters of conventional stranger homicides had been committed in a public place, as opposed to only about half of nonconventional stranger homicides, and, for the variable as a whole, a statistically significant trend was uncovered; in the latter group, however, percentages of offenses committed in a public place varied greatly, from around 60% in incidents characterized by the offender's ostensibly mentally aberrant behavior or committed in the context of a hate offense or politically motivated offense to about 20% in incidents committed in the context of a sexual offense or a mass killing or series of homicides. Incidents immediately preceding the homicide were ranked according to the degree of offender initiation. Offenders had initiated contact with the victim, including by means of surprise attack, in almost two-thirds of nonconventional stranger homicides, compared to slightly less than a third of conventional stranger homicides, a difference that was statistically significant; still, contact with the victim had been initiated by means of surprise attack in about half of homicides committed in the context of a robbery or burglary or a gangland conflict. Finally, when the method of violence was ranked by degree of intimacy, distributions of frequencies did not differ significantly between conventional-stranger-homicide and nonconventional-stranger-homicide incidents; however, whereas the use of a knife, axe or other sharp object was the most common method in both groups—occurring in about 45% of conventional stranger homicides and about 53% of nonconventional stranger homicides—the second most common method of violence was, in the former group, battery (with or without a blunt object) and, in the latter group, strangulation, suffocation or drowning.

### Offender characteristics

In Tables 1 and 2, offender characteristics are presented for 164 conventional stranger homicides and 60 nonconventional stranger homicides committed by male offenders; in the former group, homicides are subcategorized by precipitating event, and in the latter group by distinguishing feature. The median age of offenders was three years higher in nonconventional stranger homicides than in conventional stranger homicides—26 years versus 23 years—but the distribution of ranked ages did not differ significantly between groups. The percentage of offenders born abroad was also similar, at about 35% in both groups. In about 72% of conventional stranger homicides, however, offenders had been intoxicated, compared to about 44% in nonconventional stranger homicides, a difference that was statistically significant. A similar percentage of offenders in each group, about 55%, had been apprehended within 48 h of the police becoming aware of the incident. The majority of offenders in both groups had at least one previous criminal conviction, with a higher percentage in conventional-stranger-homicide offenders than in nonconventional-stranger-homicide offenders (80.1% versus 62.5%); the difference, however, was not statistically significant. Finally, the percentage of offenders adjudged to be suffering from a severe mental disorder was markedly – and significantly – higher in nonconventional-stranger-homicide offenders than in conventional-stranger-homicide offenders (43.3% versus 2.4%), and highest in the subcategory of offenders who had displayed ostensibly mentally aberrant behavior at the time of the offense (76.9%).

## Discussion

In the present study, using data from a nationwide registry, we analyzed crime scene and offender characteristics in 224 unique stranger-homicide incidents. Based on the presence of a commonplace motive, 164 incidents were categorized as conventional stranger homicides and subcategorized according to precipitating events. Regardless of the precipitating event, the majority of these homicide incidents were committed in a public place during the evening or night and involved male victims; moreover, offenders—who, on average, were in their 20s—had, in a majority of cases, a prior criminal conviction and were virtually never adjudged to be suffering from a severe mental disorder. At the same time, subcategories differed notably with regard to seven crime-scene and offender characteristics. First, victims of homicides committed in the context of a robbery or burglary were, on average, 50 years old—20 years older than victims in the other subcategories. Second, victims of homicides committed in gangland conflicts had more often been born abroad than victims in other subcategories. Third, victims of homicides resulting from a spontaneous altercation were more often intoxicated at the time of the offense than victims in other subcategories. Fourth, homicides resulting from a spontaneous altercation were also typically triggered by a mutual conflict between victim and offender, whereas, homicides occurring in the context of a robbery or burglary were typically initiated by surprise attack; in homicides committed in the context of a gangland conflict, a mutual conflict and surprise attack each accounted for half of the antecedent incidents. Fifth, a majority of homicides committed in the context of a gangland conflict were perpetrated using a firearm, whereas homicides in the other two subcategories were typically committed with a knife, axe or other sharp object. Sixth, homicides occurring in the context of a robbery or burglary or a gangland conflict were more often committed by offenders born abroad than homicides resulting from a spontaneous altercation. Seventh, offenders were intoxicated at the time of the offense in about two-fifths of homicides committed in the context of a gangland conflict; in about three-fifths of homicides committed in the context of a robbery or burglary; and in no fewer than about four-fifths of homicides resulting from a spontaneous altercation. The generalizability of the above observations remains unknown, as the comparisons are beyond the stated scope of the current study and have not been subjected to formal significance testing. Yet, several observations appear to be in line with the results of prior research. For example, in a recent study from Japan, 37.3% of victims in stranger homicides with money-oriented motives were 60 years of age or older, compared to 23.3% of victims in stranger homicides without money-oriented motives^4^; and in a recent analysis of the increase in gun violence among males in Sweden during the years 1996 to 2015, the authors point out that younger victims of lethal and non-lethal gun violence had often been involved in organized crime, and that, even in metropolitan areas, shootings mainly occurred in disadvantaged neighborhoods with a high proportion of residents with immigrant backgrounds.^18^

Based on the absence of a commonplace motive, 60 incidents were categorized as nonconventional stranger homicides and subcategorized according to unusual distinguishing features. (One subcategory—homicides committed in the context of a mass killing or series of homicides—consisted of only five incidents; therefore, here, discussion of the group's features will mostly focus on the remaining three subcategories.) Nonconventional stranger homicides turned out to be a heterogeneous group, although, in general, they were committed by men in their 20s with a previous criminal conviction, one-third to one-half of whom had been born abroad. Only in the subcategory of homicides characterized by the offender's ostensibly mentally aberrant behavior were the majority of incidents committed on weekdays or during the day. Moreover, homicides characterized by the offender's ostensibly mentally aberrant behavior or committed in the context of a sexual offense were never perpetrated with an accomplice, as opposed to about one-third of homicides committed in the context of a hate offense or politically motivated offense. In addition, victims of homicides committed in the context of a sexual offense were in all cases females, with an average age of 27 years; whereas, in the other subcategories, at least half the victims were 35 years of age or older, with the majority being males. Further, in homicides characterized by the offender's ostensibly mentally aberrant behavior, only about one-fifth of victims were intoxicated at the time of the offense, compared to almost half of the victims in the other subcategories taken together; conversely, almost half of homicides characterized by the offender's ostensibly mentally aberrant behavior were committed in a public place in the presence of witnesses, compared to about one-fifth of incidents in the other subcategories taken together. Further, in about three-quarters of the homicides characterized by the offender's ostensibly mentally aberrant behavior, offenders were apprehended within two days of the police becoming aware of the offense, compared to about half of offenders in homicides committed in the context of a hate offense or politically motivated offense and about a quarter of offenders in homicides committed in the context of a sexual offense. Finally, upon forensic-psychiatric evaluation, only about a fifth of offenders in the last two subcategories taken together were adjudged to be suffering from a severe mental disorder, compared to no fewer than about three-quarters of offenders in homicides characterized by the offender's ostensibly mentally aberrant behavior. As above, the generalizability of these observations remains unknown, as the comparisons are beyond the scope of the study and have not been subjected to formal significance testing.

Upon revision of the Swedish penal code in 1992, the legal concept of severe mental disorder was introduced to describe psychiatric conditions on the basis of whose seriousness sufferers could, according to the law, be involuntarily incarcerated.^19^ In principle, the concept includes conditions such as severe dementia and depression with suicidal ideation, as well as certain personality disorders and intellectual disabilities; in practice, however, in criminal cases, the majority of offenders who, owing to the presence of a severe mental disorder, are sentenced to forensic-psychiatric care will have been found to be suffering from either a chronic psychotic condition, such as schizophrenia, or a disabling form of autism. A number of previous studies have explored crime-scene and offender characteristics in homicides committed by offenders with a mental illness; however, in a survey of five of these studies,^20–24^ complemented by the authors’ own results, Richard-Devantoy and colleagues^25^ reported that the percentage of homicides in which the offender and victim were unacquainted was generally low, ranging from 2% to 18%. To our knowledge, only one previous study has attempted to characterize mentally ill perpetrators of stranger homicide: in that study,^26^ however, characteristics of psychotic offenders who had killed a stranger were compared with those of other psychotic offenders, who, instead, had killed a relative, rather than to those of stranger-homicide offenders in whom severe mental illness was absent.

In the present study, our ambition was not explicitly to characterize mentally ill stranger-homicide offenders. Still, informed by the archetypal construct of stranger homicide found in the literature, as well as the notion of “act of madness” used in the Swedish popular press, we hypothesized that nonconventional-stranger-homicide offenders would more often be found to be suffering from a severe mental disorder than conventional-stranger-homicide offenders. In the event, the hypothesis was convincingly confirmed: upon forensic-psychiatric evaluation, 43.3% of nonconventional-stranger-homicide offenders were adjudged to be suffering from a severe mental disorder, compared to 2.4% of conventional-stranger-homicide offenders. At the same time, the former group turned out to be strikingly heterogeneous, with frequencies of severe mental disorder ranging, in the component subcategories, from 76.9% in homicides characterized by the offender's ostensibly mentally aberrant behavior to 17.6% in the other three subcategories taken together. Our second hypothesis—that nonconventional stranger homicides offenses would be more often initiated by the offenders themselves than conventional stranger homicide offenses—was also confirmed. Indeed, when both variables are considered together, it becomes apparent that these hypotheses primarily held for the subcategory of nonconventional stranger homicides characterized by the offender's ostensibly mentally aberrant behavior: here, in 50% of incidents, offenses were committed by means of a surprise attack by offenders who ultimately were adjudged to be suffering from a severe mental disorder, compared to in only 2.2% of incidents in the other six subcategories taken together (results not shown in either table).

## Methodological considerations

The present study has a number of limitations. First, for obvious reasons, we were unable to study offender characteristics in homicides in which the offender had not been identified: indeed, the designation “stranger homicide” presupposes, by definition, characterization of the offender's relation to the victim and, thus, identification of the offender. Second, the hierarchical assignment of each homicide incident to a single subcategory was, as described above, performed by the Swedish National Council for Crime Prevention, and, upon retrieval of data from the council's registry, no information regarding the reliability of the assignment process was provided to the authors. Third, owing to the limited number of offender-related variables contained in the registry—as well as their crudely demographic nature—we were unable to analyze potentially interesting features of the offenses and offenders, for example, the distance between the offender's home and the site of the incident, or specific diagnoses rendered in connection with forensic-psychiatric evaluations. Fourth, owing to the well-documented increase in gangland homicides in Sweden during the past few years,^2^ it is likely that our results do not reflect current conditions, particularly with regard to conventional stranger homicides. Fifth, as outlined above, within each of the compared groups—conventional stranger-homicide incidents and nonconventional stranger-homicide incidents—heterogeneity was observed, regarding several variables, across component subcategories, arguably undermining the generalizability of conclusions drawn from comparisons of the two groups in the present sample.

At the same time, this study has a number of strengths. For example, the incidents included in the study were retrieved from a nationwide registry containing all homicides committed in Sweden during a 24-year period, resulting in a large dataset. In addition, our reformulation of traditional stranger-homicide typology, based on the presence or absence of a commonplace motive, allowed us to identify a subcategory of nonconventional stranger homicides—those characterized by the offender's ostensibly mentally aberrant behavior, half of which were committed by mentally ill offenders by means of a surprise attack—whose features, to a large extent, corresponded to those captured by both the archetypal stranger-homicide construct and the popular notion “act of madness.”

In conclusion, conventional stranger homicides, defined by the presence of a commonplace motive, were more often committed with an accomplice and against a male victim than nonconventional stranger homicides, defined by the absence of such a motive. By contrast, in the latter group, offenders more often initiated the contact that preceded the offense, often by means of a surprise attack. Finally, whereas nonconventional-stranger-homicide offenders were less often intoxicated at the time of the offense than conventional-stranger-homicide offenders, they were, upon forensic–psychiatric evaluation—by a very wide margin—more often adjudged to be suffering from a severe mental disorder.

## Highlights

Stranger violence has been described as one of the most frightening forms of criminal victimization.

The archetypal stranger-homicide construct resembles the FBI's notion of “motiveless homicide.”

Here, the notion of “act of madness” captured homicides by ostensibly mentally aberrant offenders.
